# Hospital Saturday Fund

**Published:** 1906-04-14

**Authors:** 


### HOSPITAL SATURDAY FUND.

The thirty-second annual general meeting of the Hospital Saturday Fund Association was held on Saturday evening, at the offices, Gray's Inn Road. Sir Savile B. Crossley (the Chairman of the fund) presided.

The report of the Council for 1905, which was adopted, showed that the receipts from the workshops and business houses had reached £25,930, as compared with £24,344 in 1904. The total was the highest recorded in the history of the movement. The Board of delegates in January last distributed the sum of £23,631 among 202 hospitals, dispensaries, and convalescent homes, this amount being £1,766 more than in any former year. The Council were glad to note that the National Association for the Establishment and Maintenance of Sanatoria for Workers suffering from Tuberculosis was making satisfactory progress. A site of 252 acres at Benenden, in Kent, had been secured, and building operations will shortly commence. The fund have voted the sum of £500 towards this work, and £300 have been paid. The thanks of the Council were accorded to the London Press for its uniform courtesy and assistance during 1905.

At the close of the annual meeting the ordinary quarterly meeting was held. The Finance Committee reported that the receipts from January 9 to March 17 had reached £2,506, as compared with £2,674 at the corresponding period of last year.

